# High flow nasal oxygen after bariatric surgery (OXYBAR), prophylactic post-operative high flow nasal oxygen versus conventional oxygen therapy in obese patients undergoing bariatric surgery: study protocol for a randomised controlled pilot trial

**DOI:** 10.1186/s13063-018-2777-2

**Published:** 2018-07-27

**Authors:** Rachel Fulton, Jonathan E. Millar, Megan Merza, Helen Johnston, Amanda Corley, Daniel Faulke, Ivan Rapchuk, Joe Tarpey, Philip Lockie, Shirley Lockie, John F. Fraser

**Affiliations:** 1 Critical Care Research Group, The Prince Charles Hospital, Rode Road, Brisbane, QLD 4032 Australia; 2 Faculty of Medicine, University of Queensland, Brisbane, Australia; 3 Wellcome-Wolfson Centre for Experimental Medicine, Queen’s University Belfast, Belfast, Northern Ireland UK; 4 St Andrews War Memorial Hospital, Brisbane, Australia; 5 Griffith University, Griffith, Queensland Australia

**Keywords:** Obesity, Bariatric surgery, High flow nasal oxygen, Post-operative respiratory complications, Electrical impedance tomography

## Abstract

**Background:**

The incidence of obesity is increasing worldwide. In selected individuals, bariatric surgery may offer a means of achieving long-term weight loss, improved health, and healthcare cost reduction. Physiological changes that occur because of obesity and general anaesthesia predispose to respiratory complications following bariatric surgery. The aim of this study is to determine whether post-operative high flow nasal oxygen therapy (HFNO_2_) improves respiratory function and reduces the incidence of post-operative pulmonary complications (PPCs) in comparison to conventional oxygen therapy in these patients.

**Method:**

The OXYBAR study is a prospective, un-blinded, single centre, randomised, controlled pilot study. Patients with body mass index (BMI) > 30 kg/m^2^, undergoing laparoscopic bariatric surgery, will be randomised to receive either standard low flow oxygen therapy or HFNO_2_ in the post-operative period. The primary outcome measure is the change in end expiratory lung impedance (∆EELI) as measured by electrical impedance tomography (EIT). Secondary outcome measures include change in tidal volume (∆Vt), partial arterial pressure of oxygen/fraction of inspired oxygen (PaO_2_/FiO_2_) ratio, incidence of PPCs, hospital length of stay and measures of patient comfort.

**Discussion:**

We hypothesise that the post-operative administration of HFNO_2_ will increase EELI and therefore end expiratory lung volume (EELV) in obese patients. To our knowledge this is the first trial designed to assess the effects of HFNO_2_ on EELV in this population. We anticipate that data collected during this pilot study will inform a larger multicentre trial.

**Trial registration:**

Australian New Zealand Clinical Trials Registry (ANZCTR), ACTRN12617000694314. Registered on 15 May 2017.

**Electronic supplementary material:**

The online version of this article (10.1186/s13063-018-2777-2) contains supplementary material, which is available to authorized users.

## Background

Obesity, defined as a body mass index (BMI) > 30 kg/m^2^, has almost doubled since 1980, with more than 671 million people worldwide now classified as obese [1, 2]. Health problems associated with obesity impact on quality of life and impose a significant cost burden to health services. Being obese is associated with a higher frequency of cardiovascular disease [3], of metabolic diseases such as type II diabetes mellitus [4], of respiratory morbidity secondary to obstructive sleep apnoea (OSA) and obesity hypoventilation syndrome (OHS) [5]. In Australia, health expenditures of those with a BMI between 30 and 35 kg/m^2^ are 19% higher than those of a normal-weight individual. This increases to 51% in those with a BMI > 35 kg/m^2^ [6]. Overall, the total financial cost of obesity is estimated to be 8.3 billion AUD [7].

Obesity is difficult to treat. Diet, exercise and medications are only modestly effective in aiding weight loss [8]. In selected individuals, bariatric surgery may offer a means of achieving long-term weight loss, improved health outcomes and a reduction in healthcare spending [9–11]. However, bariatric procedures are complicated by the additional risks associated with anaesthesia and surgery in the obese patient. Obesity and its associated co-morbidities, have been shown to increase the rate of post-operative myocardial infarction, peripheral nerve injury, wound and urinary tract infection and the requirement for re-intubation [12].

The evidence that obesity increase the incidence of PPCs is mixed. A retrospective observational study by Baltieri et al. described a 37% prevalence of atelectasis in obese patients following bariatric surgery [13]. Several studies have identified obesity as an independent risk factor for post-operative respiratory complications [14–17], while others have failed to find an association [18–20]. However, respiratory complications are not infrequent amongst the general surgical population and have been shown to increase hospital length of stay and mortality [21].

Physiological and pathological changes that occur because of obesity adversely affect both lung mechanics and gas exchange. First, there is a reduction in total respiratory system compliance and an increase in airway resistance [22, 23]. Increased mass loading on the chest wall, cephalad displacement of the diaphragm and an increase in pulmonary blood flow contribute to this [24, 25]. Taken together, these changes lead to higher work of breathing [22] and a tendency toward shallow, rapid tidal volumes (Vt) [26]. Second, breathing at a lower Vt leads to a reduction in end expiratory lung volume (EELV) [27], increasing the potential for atelectasis and ventilation/perfusion mismatching [28, 29]. The physiological alterations outlined above are further exacerbated when the patient is in the supine position and by general anaesthesia (GA), with functional residual capacity (FRC) falling by up to 50% on induction [30]. This reduction persists longer into the post-operative period in obese compared to non-obese individuals [16].

### High flow nasal oxygen therapy

High flow nasal oxygen (HFNO_2_) therapy was first shown to be an effective treatment for acute respiratory failure in the paediatric and neonatal populations [31, 32]. Recently, it has gained popularity as a therapy in adult patients, with an expanding list of clinical applications [33].

High flow nasal cannulae (HFNC) are designed to deliver an air/oxygen blend at a predetermined fraction of inspired oxygen (FiO_2_). The heating and humidification of inspired gases allows for higher flow rates to be tolerated when compared to conventional oxygen delivery devices. Flow rates of up to 70 L/min can be achieved. The process of heating and humidification also reduces mucosal drying, improves muco-ciliary clearance and reduces energy expenditure and work of breathing [34]. Higher flow rates reduce room air entrainment during inspiration and flush expired air from the upper airway during expiration, leading to the delivery of higher and more consistent FiO_2_ [34]. There is additional evidence to suggest that the higher flow rates achievable with HFNC generate a degree of positive airway pressure, which increases end expiratory lung volume (EELV) and alveolar recruitment [35, 36].

### Electrical impedance tomography

Electrical impedance tomography (EIT) is a non-invasive, radiation-free, functional imaging modality [37]. It uses changes in bio-impedance across lung tissue during the respiratory cycle to provide information on ventilation distribution, lung volumes, and regional lung mechanics [38]. EIT has been successfully validated against several other imaging and measurement modalities and strong linear correlation between the change in end expiratory lung impedance (EELI) and the EELV has been described [39–42].

### Rationale for the study

A rise in the incidence of obesity has resulted in a rapid escalation in the number of bariatric procedures being carried out worldwide. Obese patients undergoing surgery of any kind are at a higher risk of developing post-operative atelectasis secondary to the physiological and pathological changes outlined above. Study data from our group suggests that the use of HFNO_2_ when compared to low-flow oxygen devices significantly increases EELI (which in turn is associated with an increase in EELV). This relationship appears to be enhanced at higher BMIs. In the study of Corley et al., a mean increase in EELI of 1517 ± 46.6 units was associated with a reduction in respiratory rate, an increase in P/F ratio, and a reduction in a standardised dyspnoea score [35].

We propose to carry out a randomised, controlled pilot study to evaluate the effects of post-operative HFNO_2_ therapy on EELV in the obese population undergoing laparoscopic weight reduction surgery. Data gathered will be used to explore the mechanism of action of HFNO_2_ therapy in this setting and to inform the design of a larger trial with a patient-centred primary outcome.

## Methods/design

### Objectives and design

#### Research hypothesis

In obese adult patients undergoing laparoscopic bariatric surgery, prophylactic post-operative HFNO_2_ therapy will increase EELI and therefore EELV, improve respiratory function and reduce the incidence of PPCs.

#### Study design

**Fig. 1:**
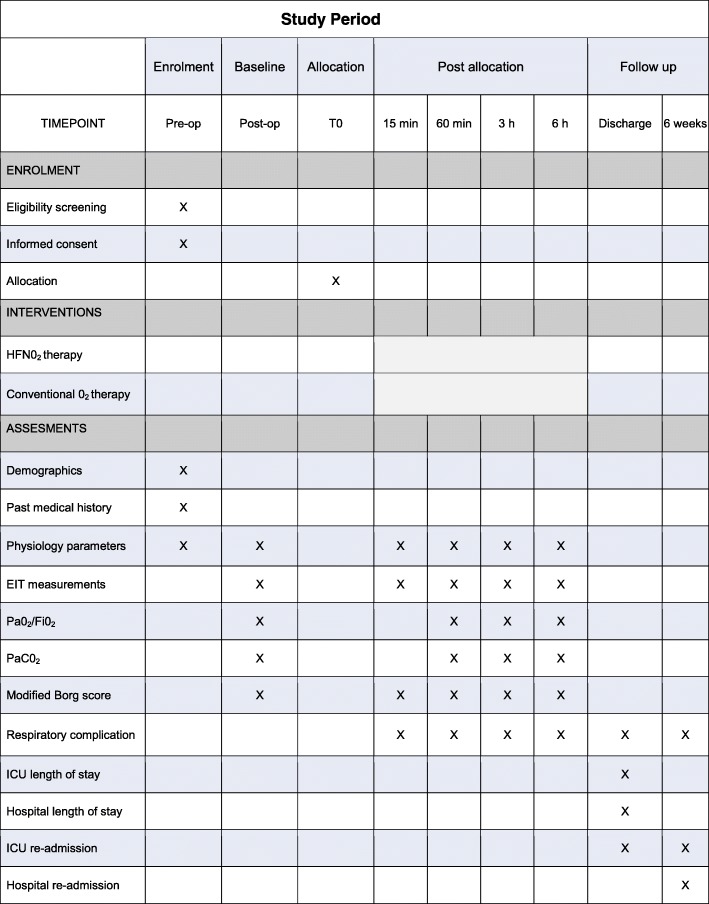
A schedule for enrolment, intervention and assessment (Standardised Protocol Items: Recommendations for Interventional Trials (SPIRIT) figure). HFNO_2_, high flow nasal oxygen; EIT, electrical impedance tomography; PaO_2_/FiO_2_, arterial partial pressure of oxygen/fraction of inspired oxygen; PaCO_2_, arterial partial pressure of carbon dioxide

This is a prospective, un-blinded, single centre, randomised, controlled pilot study, with an allocation ratio of 1:1. This protocol has been designed in accordance with the Standardised Protocol Items: Recommendations for Interventional Trials (SPIRIT) guidelines and checklist [43] (Additional file 1). A schedule for enrolment, intervention and assessment (SPIRIT figure) is outlined in Fig. 1.

#### Study objectives

The primary objective of this study is to determine the effect of post-operative HFNO_2_ on EELV in obese patients undergoing bariatric surgery. The primary outcome measure is ∆EELI between baseline and 6 h post allocation.

Secondary objectives are to determine the effect of HFNO_2_ on oxygenation and respiratory parameters, the incidence of PPCs, hospital length of stay and patient comfort. In addition, this study will provide important data (protocol feasibility, estimates of post-operative complications) and experience (consent and screening procedures, refining data collection processes), which will be used to design a large trial powered to detect differences in patient centred outcomes.

#### Study setting

The OXYBAR study will take place at St Andrews War Memorial Hospital (SAWMH), Brisbane, Australia. The hospital provides tertiary level care in a 250-bed facility, with a 15-bed Intensive Care Unit (ICU).

#### Sample size and study duration

This is a pilot study and as such no formal power calculation has been made. A convenience sample of 50 patients has been chosen based on anticipated recruitment rates and a feasible study duration.

Given our institution’s current workload, approximately two patients per week will meet the inclusion/exclusion criteria. Allowing for a 50% consent rate, and 10% loss to long-term follow up, recruitment could be completed in 54 weeks. Given the final follow up occurs 6 weeks post-operatively, data collection may be completed by week 60. Data analysis and write up of results will take an additional 12 weeks. Therefore, completion of the study is expected within an 18-month time frame.

#### Allocation

Randomisation will be achieved using a computer-generated random number table, with an allocation ratio of 1:1. Allocation concealment will be maintained using sequentially numbered sealed opaque envelopes. Envelopes will be prepared by an independent person. Each envelope will contain a unique patient identifier code and will allocate the participant to either the intervention group (HFNO_2_) or the control group (standard oxygen therapy). Randomisation will occur post-operatively on admission to the ICU. The theatre team will be blinded to the allocation. Due to the research design, neither the individual collecting data nor the patient can be blinded to treatment allocation.

### Participants, interventions and outcomes

#### Screening and consent

Patients opting to undergo laparoscopic surgery for weight reduction will be identified by the surgical team in the surgical outpatient clinic. Those that meet the eligibility criteria outlined in Table 1 will be given written information regarding the study at this time.

**Table 1:** Eligibility criteria

Inclusion criteria	Exclusion criteria
Age ≥ 18 years	Age < 18 years
BMI ≥ 30 kg/m^2^	Refusal to consent
Undergoing a laparoscopic procedure for weight reduction	Any contraindication to HFNO_2_
Informed consent obtained	Chest circumference too large for EIT belt
	Severe chronic lung disease
	Preoperative hypoxia SpO_2_ < 92%

*BMI* body mass index, *HFNO*_*2*_ high flow nasal oxygen, *EIT* electrical impedance tomography, *SpO*_*2*_ peripheral oxygen saturation

On the morning of surgery, patients will be approached by a member of the study team and informed written consent will be sought. The right of the patient to refuse to participate without giving reasons will be respected. All participants are free to withdraw at any time from the study without giving a reason and without prejudicing further treatment.

#### Pre-operative management

Pre-operative management of the patients in both groups will be consistent with current surgical best practice and at the discretion of the responsible surgeon. The study period will begin after informed consent is secured.

#### Intra-operative management

The method and conduct of general anaesthesia will be at the discretion of the responsible anaesthetist. Given the nature of the surgery all patients will undergo endotracheal intubation and mechanical ventilation.

When deemed appropriate by the anaesthetist, all patients will be extubated in theatre and receive supplemental oxygen at a flow rate of 6 L/min, via a Hudson mask. They will then be transferred to the ICU. On arrival in the ICU all patients will have a baseline EIT file recorded and an arterial blood gas sample will be taken (if an arterial line is present). Patients will then be randomised to either the control or intervention arm.

It is not expected that intra-operative management of recruited patients will differ significantly between study groups as all surgeries and GAs will be performed by a single surgeon (PL) and one of two anaesthetists (DF and IR).

#### Control group

The control group will continue to receive supplemental oxygen. Oxygen will be titrated to maintain peripheral oxygen saturations (SpO_2_) ≥ 95%. If, after an initial period of stabilisation in the ICU, patients consistently achieve or exceed the SpO_2_ target, therapy may be titrated downwards (including the use of conventional nasal cannulae). A minimum of 2 L/min of supplemental oxygen will be maintained for the duration of the study period.

#### Intervention group

Patients will be managed with supplemental oxygen delivered via HFNC (Airvo™ 2, Fisher & Paykel, New Zealand) at an initial FiO_2_ of 0.5 and a flow rate of 50 L/min. Oxygen therapy will then be titrated to maintain SpO_2_ ≥ 95%. A constant flow rate of 50 L/min will be maintained for the duration of the study period.

#### Outcome measures

Primary and secondary outcome measures are summarised in Table 2. The primary outcome measure is ∆EELI from baseline to 6 h post-return to ICU.

**Table 2:** Summary of primary and secondary outcome measures

Phase	Outcome measure	Timing	
Day 0	Change in end expiratory lung impedance (∆EELI)	6 h	Primary outcome
Day 0	Change in end expiratory lung impedance (∆EELI)	15 min, 60 min, and 3 h	Secondary outcomes
	Change in Tidal impedance variation (∆VARt)	0, 60 min, 3 h, and 6 h	
	Change in oxygenation (PaO_2_/FiO_2_)		
	Change in arterial CO_2_ (PaCO_2_)		
	Change in respiratory rate		
	Change in modified Borg dyspnoea score		
	Requirement for escalation of O_2_ therapy		
Discharge	Length of ICU stay	Day 2–3	
	Re-admission to ICU		
	Length of hospital stay		
	Diagnosis of a respiratory complication		
Follow up	Hospital re-admission	Week 6	
	Diagnosis of a respiratory complication		

*EELI* end expiratory lung impendence, *PaO*_*2*_*/FiO*_*2*_ arterial partial pressure of oxygen/fraction of inspired oxygen; *PaCO*_*2*_ arterial partial pressure of carbon dioxide

### Data collection, quality and management

#### Time points

Pre-operative refers to the period after recruitment, but before surgery and before randomisation. Pre-operative data collected include:

- Patient demographics (age, gender, height and weight)
- Modified Borg dyspnoea score of 0 = no dyspnoea to 10 = maximal dyspnoea [44]
- Co-morbidity data (including smoking history)
- Baseline physiological parameters (heart rate, blood pressure, respiratory rate, peripheral oxygen saturation)

Baseline refers to the period after surgery and before randomisation. All patients will return from theatre after being extubated by the responsible anaesthetist and after receiving 6 L/min of 0_2_ via a Hudson mask. Baseline data collected include:

- EIT file recording
- Arterial blood gas analysis
  - ▪ Arterial partial pressure of oxygen (Pa0_2_)
  - ▪ Arterial partial pressure of carbon dioxide (PaC0_2_)
  - ▪ Calculated Pa0_2_/Fi0_2_ ratio
- Physiological parameters (as above)
- Modified Borg dyspnoea score (as above)
- Pain score
  - ▪ Numerical scale where 0 = no pain and 10 = maximal pain
- Operative data
  - ▪ Type of operation preformed
  - ▪ Duration of operation
  - ▪ Any related complications
- General anaesthesia data
  - ▪ Type of anaesthesia administered
  - ▪ Amount of analgesia given
  - ▪ Any related complications

After baseline data have been recorded, randomisation will occur - T0 refers to this point.

T15, T60 and T3 refer to 15 min, 60 min, and 3 h after T0, respectively. Data collected include:

- EIT file recording
- Arterial blood gas analysis
- Physiological parameters
- Modified Borg dyspnoea score
- Pain score

T6 refers to 6 h after T0. Data collected include all data from the above time point in addition to:

- Data on clinical course, need for oxygen titration or escalation of therapy

Discharge refers to the day of discharge from the hospital. Discharge data collected include:

- Hospital length of stay
- Physiological parameters
- Data on clinical course, need for oxygen titration or escalation of therapy
- ICU re-admission
- Data on the incidence of PPCs

PPCs are defined in this study as any pulmonary abnormality, disease or dysfunction that adversely affects the clinical course of a patient in the post-operative period. This includes mild, moderate or severe respiratory failure, acute respiratory distress syndrome (ARDs) new onset bronchospasm, new pulmonary infiltrates on chest x-ray, pulmonary infection and the presence of new plural effusions, atelectasis, cardiopulmonary oedema or pneumothorax [45].

Six weeks refers to follow up post discharge at the surgical outpatient clinic. Six-week data collected include:

- Need for hospital re-admission
- Any respiratory complications requiring medical intervention

#### EIT data measurement

EIT measurements will be performed with the PulmoVista® 500 (Dräger Medical, Lubeck, Germany). Discreet files will be acquired over a 5-min period, with a frame rate of 20 frames/second. Files will be automatically saved to the device hard drive. Analysis will take place off-line using Dräger review software V5.1 and then downloaded using Microsoft Excel (Microsoft, Redmond, USA).

All EIT measurements will be recorded with the patient in bed inclined to 30–45°. EIT belt placement will be standardised to the 4th–5th intercostal space, below the breast tissue where appropriate. Belt position will be marked with surgical ink to ensure minimal displacement between readings.

#### Data recording

Clinical data will be recorded at the intervals described above. For routinely collected clinical data hospital notes and charts will be the source documents. For study-specific data the case report form (CRF) will be the source document.

#### Data quality

The Chief Investigator (CI)/Study Lead (SL) will provide training to site staff on trial processes and procedures. CRF completion and data collection will be the responsibility of the SL and designated team members. The clinical data management process will be governed by internal standard operating procedures (SOPs), designed to ensure adherence to International Conference on Harmonisation Good Clinical Practice (ICH GCP) guidelines.

### Statistical methods

All analyses will be undertaken by an independent biostatistician. The statistician will be blinded to treatment allocation. Analysis will be undertaken on an intention-to-treat basis. Continuous data will be checked for normality and presented as mean (standard deviation) or median (interquartile range). Categorical data will be presented as counts (percentages). Where data are continuous, they will be compared using Student’s *t* test or the Mann-Whitney U test as appropriate. Categorical variables will be compared using the chi-squared test. All tests will be two-tailed, and a *p* value <0.05 will be considered significant. For the primary outcome measure, change in EELI, a mixed-effects regression model will be used. The modelling methodology will follow that used in our previous trial of HFNO_2_ therapy [35].

### Adverse events (AE) and serious adverse events (SAE)

AEs and SAEs will be monitored and reported to the Human Research Ethics Committee (HREC). The AE reporting period for the trial begins at randomisation and ends 48 h after randomisation. The CI or their delegated investigator is responsible for recording and reporting of AEs observed during the study period. Events occurring because of the patient’s underlying condition will not be reported as an AE. The CI must assess severity, seriousness, causality and expectedness of any AEs in keeping with regulatory requirements. All events meeting the definition of an SAE should be notified to the CI within 24 h of occurrence. Follow-up information should be sought and submitted as it becomes available. The follow-up information should describe whether the event has resolved or persists, if and how it was treated and whether the patient continues in the study or has been withdrawn from treatment. Once received, seriousness, causality and expectedness will be confirmed by the CI (or delegated SL). SAEs will be notified to the Research Ethics Committee (REC) within 72 h.

## Discussion

HFNO_2_ has become more accessible in recent years and is now commonplace in the ICU, emergency departments and operating theatres. HFNO_2_ therapy is minimally invasive, safe and has few contraindications. We aim to test the hypotheses that respiratory support, in the form of HFNO_2_, leads to an increase in EELV, conferring a reduction in the incidence and duration of post-operative atelectasis.

HFNO_2_ provides a degree of positive airway pressure, which may aid alveolar recruitment and prevent de-recruitment [35, 36]. Corley et al. showed a significant increase in EELV in patients with respiratory compromise, treated with HFNO_2_, following cardiac surgery. This increase in EELV was greater in those with a higher BMI, suggesting a benefit in this cohort.

Trial recruitment began in April 2017 at St Andrews War Memorial Hospital, Brisbane and is anticipated to be completed by the end of April 2018. We aim to publish the results as a single manuscript. It is anticipated that the results of this study will be used to inform the operational design and feasibility of a larger randomised controlled trial.

### Trial status

This trial is ongoing and actively recruiting.

Date recruitment started, 3 April 2017.

Date of anticipated completion, 31 June 2018.

## Additional file

Additional file 1: SPIRIT 2013 checklist: recommended items to address in a clinical trial protocol and related documents. (DOCX 41 kb)

## Abbreviations

AE: Adverse event
AUD: Australian dollar
BMI: Body mass index
CI: Chief Investigator
CRF: Case report form
EELI: End expiratory lung impendence
EELV: End expiratory lung volume
EIT: Electrical impendence tomography
FiO_2_: Fraction of inspired oxygen
GA: General anaesthesia
HFNC: High flow nasal cannulae
HFNO_2_: High flow nasal oxygen
ICU: Intensive care unit
OHS: Obesity hypoventilation syndrome
OSA: Obstructive sleep apnoea
PaO_2_,: Arterial partial pressure of oxygen
SAE: Serious adverse event
SL: Study Lead
SOPs: Standard operating procedures
SPIRIT: Standardised Protocol Items: Recommendations for Interventional Trials
SpO2: Peripheral oxygen saturation
Vt: Tidal volume
